# Pregnancy outcomes in pregnant women taking oral probiotic undergoing cerclage compared to placebo: two blinded randomized controlled trial

**DOI:** 10.1186/s12884-024-06496-x

**Published:** 2024-05-09

**Authors:** Raziyeh Vanda, Tahora Dastani, Seyed-Abdolvahab Taghavi, Hossein Sadeghi, Nicky Lambert, Fatemeh Bazarganipour

**Affiliations:** 1 Gynecologic and Obstetrics Department, School of Medicine, Yasuj University of Medical Sciences, Yasuj, Iran; 2 Medicinal Plants Research Center, Yasuj University of Medical Sciences, Yasuj, Iran; 3 Department of Mental Health and Social Work, Middlesex University, London, UK

**Keywords:** Cerclage, Cervical insufficiency, Probiotic, Pregnancy

## Abstract

**Aim:**

The purpose of this study is to evaluate the oral probiotic effect on pregnancy outcomes in pregnant women undergoing cerclage compared to placebo.

**Methods:**

This study was a double-blind randomized clinical trial undertaken in Yasuj, Iran. 114 eligible participants who have undergone cerclage were randomly divided to either receive probiotic adjuvant or 17α-OHP (250 mg, IM) with placebo from the 16th -37th week of pregnancy by “block” randomization method. Our primary outcomes were preterm labor (PTB) (late and early) and secondary outcomes were other obstetrical and neonatal outcomes included preterm pre-labor rupture of membranes (PPROM), pre-labor rupture of membranes (PROM), mode of delivery, and neonatal outcomes including anthropometric characterize and Apgar score (one and fifth-minute).

**Results:**

Results show that there are no statistically significant differences between the two groups in terms of PTB in < 34th (15.51% vs. 17.86%; *P* = 0.73) and 34-37th weeks of pregnancy (8.7% vs. 16.1%; *P* = 0.22), and mode of delivery (*P* = 0.09). PPROM (8.7% vs. 28.5%; *P* = 0.006) PROM (10.3% vs. 25%; *P* = 0.04) was significantly lower in patients receiving probiotic adjuvant compared to the control group. After delivery, the findings of the present study showed that there were no significant differences in newborn’s weight (3082.46 ± 521.8vs. 2983.89 ± 623.89), head circumstance (36.86 ± 1.53vs. 36.574 ± 1.52), height (45.4 ± 5.34 vs. 47.33 ± 4.92) and Apgar score in one (0.89 ± 0.03 vs. 0.88 ± 0.05) and five minutes (0.99 ± 0.03vs. 0.99 ± 0.03) after birth.

**Conclusion:**

Our result has shown that the consumption of Lactofem probiotic from the 16th week until 37th of pregnancy can lead to a reduction of complications such as PPROM and PROM.

## Introduction

Cervical insufficiency is defined as the inability of the cervix to retain the products of pregnancy in the second trimester (14-27th weeks of pregnancy) and asymptomatic labor, i.e. quickly and without contractions [1]. The incidence of cervical insufficiency is less than 1% of the pregnant population [2, 3]. The diagnosis is based on three methods: firstly, on the patient’s history (two or more births less than 37 weeks or abortion in the second trimester, (which usually occur rapidly and without physical pain). It can be based on an ultrasound of the length of the cervix, as the length of the cervix in women with a history of premature birth or abortion in the second trimester, at less than 24 weeks is less than 25^mm^. Lastly it can be determined on the physical examination who presents with dilatation and effacement and without pain and contraction (with or without fetal membranes) in 14-27th weeks of pregnancy, which is called emergency or rescue cerclage [1]. In these cases, with the diagnosis of cervical insufficiency, cerclage is performed and progesterone is started as a drug supplement in the prevention of premature birth. In emergency cerclage, it is recommended to be hospitalized at least one day before the cerclage and examined for infection and bleeding [1].

Cerclage is less successful in the presence of infection, so the effectiveness of antibiotics in reducing infection and increasing the success of cerclage and the outcome of pregnancy have been researched. However, there is still controversy around emergency cerclage as some researchers recommend the use of antibiotics before and after emergency cerclage [4].

During pregnancy, treatments that restore the natural flora and acidity of the vagina without systemic effects to other treatment methods are preferable [5]. Lactobacillus, the predominant flora of the vagina, as a gram-positive and catalase-negative bacterium, plays an important role in the reproductive system, including the vagina [6]. Types of Lactobacillus in vaginal flora include Lactobacillus acidophilus, fermentum, crispatus, jensenii [7]. The human vagina is normally lined by non-keratinized squamous epithelium. The middle and surface layers contain glycogen, which is released when the surface layers break down. Free glycogen is fermented by epithelial cells and lactobacilli and produces lactic acid and hydrogen peroxide [8] and the presence and dominance of lactobacillus in the vagina is associated with a decrease in the risk of bacterial vaginosis and urinary tract infection [9]. The protective mechanism of lactobacilli includes blocking the attachment of the pathogen to the vaginal epithelium and the production of hydrogen peroxide, which inhibits the proliferation of bacteria. Some Lactobacillus strains are able to colonize in the vagina and therefore may reduce the risk of urinary infections [7]; so it becomes important to explore whether the restoration of lactobacilli with the use of probiotics can lead to an improvement in the normal condition and improve the possibility of a healthy term pregnancy. According to the Cochrane review of probiotic oral supplementation in pregnancy at risk of preterm birth, no benefit or harm was reported, although further studies are needed in this field [10].

However, the effect of probiotic on pregnancy outcome mainly PTB is not well recognized. One study found probiotic use did not increase the rate of preterm delivery and duration of pregnancy compared with placebo [11]. One of the reviews [12] also identified no evidence that taking probiotics or prebiotics during pregnancy either increases or decreases the risk of preterm birth or other infant and maternal adverse pregnancy outcomes. In contrast, in last systematic review combination of vaginal probiotics and antibiotic prophylaxis has been shown to effectively improve perinatal outcomes in women with PPROM [13]. Moreover, another study found oral probiotics containing Clostridium had a significant effect on the prevention of preterm birth before 32 weeks of gestation [14]. To date, PTB is a matter of debate in obstetric practice and considering the above contradictory evidence, this issue remains unsettled related to efficacy of probiotic.

The lack of an established evidence-based medication response in association with cerclage to improve pregnancy outcomes, the ambiguities around the role of antibiotics in increasing the success of cerclage remain a concern. When considered in combination with the potential advantages of probiotics (bacteriotherapy and immune regulation); it was evident that this study was required in order to evaluate the oral probiotic effect on pregnancy outcomes in pregnant women undergoing cerclage compared to placebo. The primary objective of this study was to investigate the efficacy of oral probiotic in preventing PTB in pregnant women undergoing cerclage.

## Methods

### Participant

This study was a randomized clinical trial undertaken in Shahid Mofateh subspecialty educational polyclinic, Yasuj, Iran between 2020 and 2022. All procedures in the current study were in accordance with the ethical standards of Yasuj University of Medical Sciences with the 1964 Helsinki declaration. The Ethics Committee of the Yasuj University of Medical Sciences, Yasuj, Iran, approved the study, by reference number: IR.YUMS.REC.1399. 130.Written informed consent was obtained from all participating. This study was registered prospectively at the Iranian Registry of Clinical Trials (www.irct.ir). Trial registration: IRCT20160524028038N11 (29/04/2021), https://www.irct.ir/trial/55864.

Inclusion criteria: age 18–45 years, gestational age between 14-24th weeks of pregnancy, cerclage by McDonald method, absence of syphilis, gonorrhea and HIV clinically single pregnancy, lack of maternal insulin-dependent diabetes mellitus, treatment of hypertension, lupus no clinical chorioamnionitis. Exclusion criteria: declined to participate in the study, non-completion of the course of treatment or use of probiotics, taking drugs that affect the intestinal microbial flora, such as antibiotics, occurrence of any genital or urinary tract infection requires antibiotic treatment during treatment.

To estimate the sample size, the findings of Badehnoosh et al.‘s study (2018) [15]and the following formula were used [15]. The final sample size was estimated to be 52 people for each group (α = 0.05; β = 0.80; P1: 20%; P2:46.7%).

$$n = \frac{{{{\left( {{Z_{1 - \alpha /2}} + {Z_{1 - \beta }}} \right)}^2}\left[ {{P_1}(1 - {P_1}) + {P_2}(1 - {P_2})} \right]}}{{{{\left( {{P_1} - {P_2}} \right)}^2}}}$$

### Randomization, hidden distribution, and blindness

Randomization was done using a computer-generated table of random set numbers in permuted blocks of four (with equal numbers to intervention and comparison group within a block, with an allocation ratio of 1:1), and allocation was concealed in sealed disclosure envelopes.

### Description of intervention for both groups

In this study, 128 eligible patients who have undergone cerclage were randomly divided into two groups. 17α-OHP (Femolife™, Aburaihan Pharmaceutical Co., Tehran, Iran, 250 mg, IM) was prescribed to all participants in two groups during our Prinatalogist (RV) from 16th -37th week of pregnancy. Moreover:

Group A: Oral probiotic (500 mg lactofem capsule), which contained Lactobacillus probiotic strains[L.acidophilus(5 × 1010CFU/g), L.plantarum(1.5 × 1010 CFU/g), L.fermentum(7 × 109CFU/g) and L.Gasseri (2 × 1010CFU/g)] made by Iranian zist-takhmir Company administered orally and daily from the 16th -37th week of pregnancy.

• Group B: Placebo capsules containing starch powder are very similar to group A prepared by the Medicinal plants research center’s Laboratory, Yasuj University of Medical Sciences, similar to probiotic capsules. This capsule is prescribed according to the method described above. All interventions and participants followed for prenatal care was under the supervision of one our project’ s perinatologist (RV) in our clinic. She visited the participants every two weeks during the study to assess the recurrence of preterm labor pain, check the proper and regular use of medications and other complications. Moreover, subjects were monitored for any side effects of the treatment during the visits and the complications were recorded.

### Data collection

Demographic data, obstetrical and neonatal outcome and side effects were gathered in checklists. The content of checklist was based on previous literature and approved by eight recognized expert panel in midwifery, genecology and obstetrics.

- Demographic and reproductive information including age, BMI (weight and height were calculated by weight/ height squared (kg/m2) in all patients), education, occupation (housewife or employed), gravidity, abortion, and parity were collected.

Our primary outcomes were PTB (late and early) and secondary outcomes were other obstetrical and neonatal outcomes as below:

- Obstetrical complications include preterm prelabor rupture of membranes (PPROM), prelabor rupture of membranes (PROM), PTB (late and early), and mode of delivery (cesarean or NVD).
- Neonatal outcomes, including weight, height, and head circumstance of the newborn and Apgar score (one and five-minute).
- Side effects, i.e., fever, itching, diarrhea, vomiting, or other gastrointestinal symptoms were recorded.

### Statistical analysis

Data analysis was done using descriptive statistics (frequency, percent, mean ± SD) and a comparison of these data was performed by x^2^ and the independent t-test. We used descriptive statistics as well as the Kolmogorov–Smirnov test to analyze the distribution of data. The statistical program for social sciences (SPSS, version 21; SPSS, Chicago, IL). *P* values were set at 0.05 for all analyses. In this study, PTB was considered as the primary outcome and pregnancy related complications as the secondary outcome. There were no missing values. Therefore, no missing imputation technique was used. This manuscript is in accordance with the PRISMA guidelines for reporting randomized trials.

### Results

**Fig. 1:**
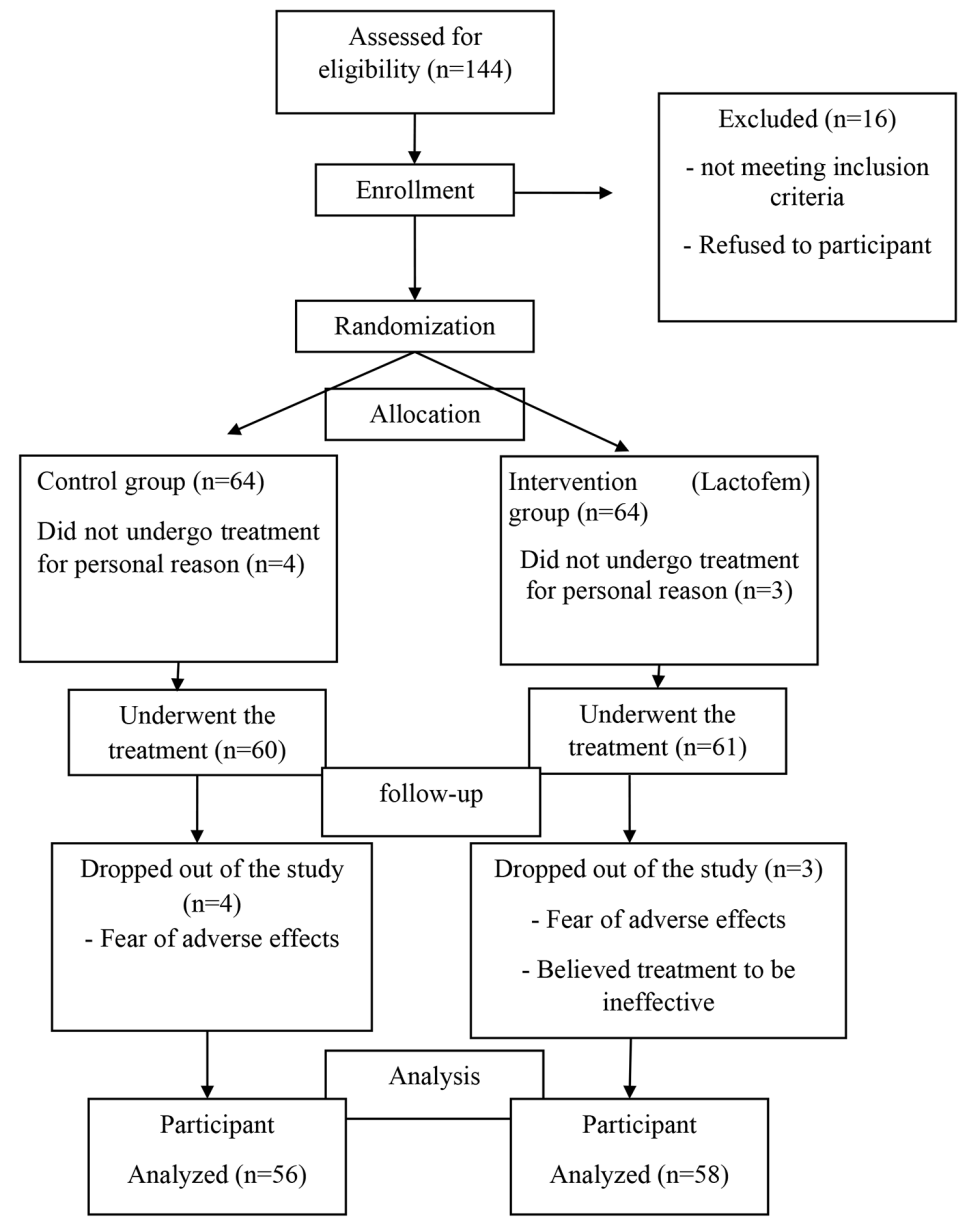
The process of allocating patients

Our annual delivery rate in Hospital (Emam Sajad Hospital, Yasuj) over the 3 years of the study was 5100. Cerclages procedure during these years performed in 180 pregnancies. It should be noted this hospital is referral and mostly high-risk pregnancies admitted. Among these, a number of 144 patients for assessed for the eligibility out of which 128 recruited patients were randomly divided into two groups (64 patients in each group). Finally, 114 patients completed the follow up (56 patients in control, and 58 patients in intervention group). The process of allocating patients is shown in Fig. 1.

### Sample characteristics

Patient characteristics are presented in Table 1. There were no statistically significant differences between the two groups according to age, BMI, education, occupation, gravidity, abortion, or parity.

**Table 1:** Clinical characteristics of the patient in study groups

Groups Variable		Probiotic group (*n* = 58)	Control group (*n* = 56)	*P* value
Age(year)*		30.07 ± 5.35	31.7 ± 5.42	0.1
Education*	High school	9(15.51)	15(36.8)	0.18
	Diploma	21(36.2)	13(23.2)	
	University	28(48.2)	28(50)	
Occupation **	Occupied	40 (68.9)	43(76.7)	0.34
	Housewife	18(31.1)	13(23.3)	
BMI (kg/m^2^) *^£^		27.41 ± 4.13	32.12 ± 7.86	0.2
Gravidity*		2.12 ± 1.6	2.25 ± 1.56	0.66
Parity *		0.27 ± 0.52	0.35 ± 0.69	0.48
Abortion*		1.5 ± 1.7	1.1 ± 1.2	0.11

*Mean ± SD; independent t test

**n (%); x^2^

^£^BMI: body mass index

### Obstetric outcomes between groups

Results show that there are no statistically significant differences between the two groups in terms of PTB in < 34th (15.51% vs. 17.86%; *P* = 0.73) and 34-37th weeks of pregnancy (8.7% vs. 16.1%; *P* = 0.22), and mode of delivery (*P* = 0.09). PPROM (8.7% vs. 28.5%; *P* = 0.006) PROM (10.3% vs. 25%; *P* = 0.04) was significantly lower in the probiotic compared to the control group. (Table 2).

**Table 2:** Obstetrics outcomes between groups

groups variable		Probiotic group (*n* = 58)	Control group (*n* = 56)	*P* value
PTB < 34th weeks of pregnancy *	yes	9 (15.51)	10(17.86)	0.73
	no	49 (84.49)	46(82.14)	
PTB in 34–37 weeks of pregnancy *	yes	5(8.7)	9(16.1)	0.22
	no	53(91.3)	47(83.9)	
PPROM^£^ *	yes	5(8.7)	16(28.5)	0.006
	no	53(91.3)	40(71.5)	
PROM ^₼^*	yes	6(10.3)	14(25)	0.04
	no	52(89.7)	42(75)	
Type of delivery *	NVD^µ^	24(41.7)	31(56.36)	0.09
	C/S^€^	31(54.44)	24(43.64)	
	instrumental	3(5.17)	0(0)	

*n (%); x^2^

^£^PPROM: Preterm pre-labor rupture of membrane; ^₼^: PROM: pre-labor rupture of membrane; ^ℓ µ^NVD: normal vaginal delivery; ^€^ C/S: cesarean section

### Neonatal outcomes between groups

After delivery, the findings of the present study showed that there were no significant differences in newborn’s weight (3082.46 ± 521.8 vs. 2983.89 ± 623.89; *P* = 0.36), head circumstance (36.86 ± 1.53 vs. 36.574 ± 1.52; *P* = 0.32), height (46.4 ± 5.34 vs. 47.33 ± 4.92; *P* = 0.15) and apgar score in one (0.89 ± 0.03 vs. 0.88 ± 0.05; *P* = 0.131) and five minutes (0.99 ± 0.03 vs. 0.99 ± 0.03; *P* = 0.138) after birth. (Table 3)

**Table 3:** Neonatal outcomes between groups

groups variable*	Probiotic group (*n* = 58)	Control group (*n* = 56)	*P* value
Newborn’s height (cm)	46.4 ± 5.34	47.33 ± 4.92	0.15
Newborn’s weight (gram)	3082.46 ± 521.8	2983.89 ± 623.89	0.36
Newborn’s head circumstance (cm)	36.86 ± 1.53	36.574 ± 1.52	0.32
Apgar score in one minute after birth	0.89 ± 0.03	0.88 ± 0.05	0.131
Apgar score in five minute after birth	0.99 ± 0.03	0.99 ± 0.03	0.138

* Mean ± SD; independent t test

### Side effects

No side events were reported in either group.

## Discussion

This study as first investigated the effect of an oral intake of probiotic, starting at the 16th -37th week of pregnancy in pregnant women undergoing cerclage compared to placebo. We found that the orally ingested probiotic that were used here, compared with placebo, did not decrease the proportion of PTB in < 34th and 34-37th weeks of pregnancy. Our data, however, are based on only a few cases and have therefore to be interpreted with caution. Like ours, all other studies that have been published to date have been too small to demonstrate effectiveness of this intervention in the prevention of PTB. One randomized controlled trial analyzed the effect of probiotics on the occurrence of PTB and failed to show any effect, possibly because of low power [11]. Moreover, one of the reviews [12] also identified no evidence that taking probiotics or prebiotics during pregnancy either increases or decreases the risk of preterm birth or other infant and maternal adverse pregnancy outcomes. Limitation of this review was the small size of primary studies cause to outcomes lacked sufficient statistical power.

Our finding was not powered to detect changes in the incidence of PTB; numerically, however, probiotics were associated with a lower PPROM; prevalence of PPROM was significantly higher in control than oral lactobacillus group. PPROM is one of the predisposing factors for PTB. Although this complication only occurs in about 3% of pregnancies, it is known to be the cause of one third of PTB [16]. Various studies have reported the relationship between systemic infections, such as genitourinary system infection and bacterial vaginosis, and an increased risk of PTB and PROM [17]. Lactobacillus species including *Lactobacillus acidophilus, Lactobacillus plantarum, Lactobacillus fermentum* and *Lactobacillus* gasseri, constitute the predominant normal microbial flora of genitourinary and gastrointestinal (GI) tract of healthy individuals. The effectiveness of these probiotics in maintenance of the normal pH of vagina and prevention of genital infections has been well-studied [18]. Host immunity modification and interference with colonization of external pathogens are considered their main mechanisms of action [19, 20]. Therefore, it is considered likely that Lactofem will reduce the PPROM by suppressing and controlling maternal infections (known and unknown). Mercer et al. showed that the use of prophylactic probiotics significantly prolongs pregnancy and reduces maternal and infant mortality [17] which is consistent to with the findings of this study. Moreover, in the retrospective study, Kirihara et al. (2018), investigated the effect of oral probiotics on perinatal outcomes in patients at risk of PTB. The probiotics were used as prophylaxis for bacterial vaginosis containing Streptococcus faecalis, Clostridium butyricum and Bacillus mesentericus. The results demonstrated that the use of probiotics as prophylaxis can increase the duration of pregnancy and prevent PTB [14]. Moreover, in our study, the PROM was higher in the control compared to the lactofem group. In contrast to our study, Badehnoosh., et al. (2018) have shown that the use of probiotics in pregnant women with gestational diabetes did not lead to improved pregnancy outcomes. However, compared to placebo, probiotic supplementation led to a slight reduction in cesarean section rates although the outcomes of pregnancy did not change significantly [15].

Whilst, Lee., et al. (2012), report that lactobacillus consumption in early pregnancy have no significant effects on spontaneous abortions, PTB, and low birth weight infants [21]. This present study, found PPROM and PROM significantly decreased in the group receiving probiotics. This difference with these findings could be influenced by factors such as study design, different doses of probiotics, and the subjects studied.

In this present study, the patients were followed up until delivery. After delivery, the findings of the present study showed that there were no significant differences in newborn’s weight, head circumstance, height and Apgar score in one and five minutes after birth. In line with the results of the present study, Lindsay, et al. (2015) also investigated the complications of pregnancy and reported there was no significant difference between the birth weight and Apgar at birth in the placebo and probiotic groups [22].

Lactobacillus species in our study including Lactobacillus acidophilus, Lactobacillus plantarum, Lactobacillus fermentum and Lactobacillus gasseri, constitute the predominant normal microbial flora of genitourinary and gastrointestinal (GI) tract of healthy individuals. The effectiveness of these probiotics in maintenance of the normal pH of vagina and prevention of genital infections has been well-studied [23]. Oral administration of 10^9^–10^11^colony-forming units (cfu) of lactobacilli in our study is the standard dose believed to be required for passage through the intestine and subsequent improvement of gut and vaginal health [23–26]. There are many variables that influence vaginal colonization by lactobacilli including glycogen level, substances used in vaginal washing, the use of antibiotics, and the ability of lactobacilli to produce substances such as hydrogen peroxide [27, 28]. An advantage of the oral route in our study is that it may reduce pathogen ascendance from the rectum to perineum and vagina, while a concern of the intravaginal approach for some women may be the more invasive instillation of microbes [29]. Furthermore, these capsules modify microbiota of urogenital and GI tract and prevent from infections by improving immune system function. Lactofem capsules are readily available in our country at a reasonable price. Another issue is strains of the probiotic source (human vs. nonhuman). To determine which probiotics should be isolated depends on the purpose of their use. Theoretically, probiotics should be isolated, from the same source as the target source to be reused. If probiotics are to be used in human, they should be isolated from human (such as human gastrointestinal tract) as probiotics isolated from human have better adhesion to the human intestinal wall and are likely to be safer than those isolated from non-human sources. Some previous study identified human Lactobacillus has better probiotic potential and application prospects than strains from the nonhuman source [30]. However, it’s proposed that despite the name, human-strain probiotics do not contain human by-products or ingredients. They are simply strains of beneficial bacteria that have been found to live in the human digestive tract. That means they are already adapted to thrive in the human gut. According previous reports, because they are native to the human intestinal tract, they are superior to probiotics from other sources. The two most prevalent types of native bacteria in your gut and in human strain probiotic dietary supplements are Lactobacilli, which are found in the small intestines, and Bifidobacterial, which reside in the large intestines [31].

Limitations of the current study include the small sample size. Due to the small sample size, the power to detect changes in the primary outcome i.e., PTB measures was low. Therefore, the current study may suffer from type two error, being underpowered to detect true differences in the reported PTB. However, the study was powered to detect a between-group difference in our secondary outcome i.e., PPROM and another pregnancy outcome (power > 80%). Strict and extensive sample inclusion and exclusion criteria make this a homogenous set of cases and controls. Furthermore, participants had diverse dietary habits and a wide range of intake frequencies of probiotic products. Sampling from a single clinic increases the possibility of selection bias and longer follow-up of babies was not possible. The dosage of lactobacillus capsules administered could be another limitation. Maybe at higher doses, more improvement in pregnancy outcome could have been resulted. Future large sample size studies with longer intervention duration are needed to confirm these findings. Another area that could yield useful data in future studies would be the evaluation of the effects of probiotic supplementation on other pregnancy outcomes, including infant respiratory status and length of stay in the neonatal intensive care unit.

## Conclusion

In conclusion, results from this study demonstrate that the consumption of Lactofem probiotic from the 16th-37th of pregnancy can lead to a reduction of complications such as PPROM and PROM. Its usefulness in the prevention of PTB, however, remains unclear. However, further RCTs are needed to investigate different species and doses for a longer duration to improve our understanding regarding the role of gut microbiota in pregnancy.

## Data Availability

The primary data for this study from the authors on direct request.

## Abbreviations

PPROM: preterm pre-labor rupture of membranes
PROM: pre-labor rupture of membranes
PTB: preterm birth
NVD: normal vaginal delivery
BMI: body mass index
